# Knee valgus proprioception, muscle activity, patient-reported outcomes and dynamic balance control after anterior cruciate ligament reconstruction: a cross-sectional case–control study

**DOI:** 10.1186/s43019-026-00341-5

**Published:** 2026-08-20

**Authors:** Mengde Lyu, Adrian Pranata, Joshua Farragher, Jinglei Huang, Huaibin Qian, Roger Adams, Weixin Zhang, Ji Sun, Qingrong Xu, Jia Han, Evangelos Pappas

**Affiliations:** 1 Jiangwan Hospital of Hongkou District, Shanghai University of Medicine and Health Sciences, Shanghai, China; 2 School of Health and Biomedical Science, STEM College, RMIT University AU, Melbourne, Australia; 3 College of Rehabilitation Sciences, Shanghai University of Medicine and Health Sciences, Shanghai, China; 4 Research Institute for Sport and Exercise, University of Canberra, Canberra, Australia

**Keywords:** Anterior cruciate ligament reconstruction, Proprioception, Electromyography, Knee valgus, Neuromuscular control

## Abstract

**Background:**

Proprioceptive deficits following anterior cruciate ligament reconstruction (ACLR) are commonly assessed using sagittal-plane, nonweight-bearing tasks that may not reflect the demands of functional activities. This study evaluated the test–retest reliability and discriminative validity of a weight-bearing knee valgus discrimination task (Knee-VEDA) and compared proprioceptive acuity, muscle activity, dynamic balance, and patient-reported outcomes between individuals with ACLR and healthy controls.

**Methods:**

Sixty-three participants (32 ACLR, 31 controls) performed the Knee-VEDA with concurrent surface electromyography (sEMG) of seven lower-limb muscles. Test–retest reliability was assessed. Dynamic balance and patient-reported outcomes were evaluated using the Y-Balance Test, International Knee Documentation Committee (IKDC) form, and Knee injury and Osteoarthritis Outcome Score (KOOS).

**Results:**

The Knee-VEDA demonstrated acceptable-to-good test–retest reliability (ACLR ICC = 0.84; controls ICC = 0.64) and acceptable discriminative validity (AUC = 0.835, *p* < 0.001). The ACLR group exhibited significantly poorer proprioception in the operated limb compared with their intact limb and controls (*p* < 0.001). sEMG analysis revealed not statistically significant between-group differences in lower-limb muscle activity. ACLR participants also demonstrated inferior Y-Balance performance compared with controls (_p_ = 0.02). In the ACLR group, better Knee-VEDA scores correlated with better Y-Balance (_r_ = 0.50, *p* < 0.001), and better KOOS symptoms scores (_r_ = 0.55, _p_ = 0.001).

**Conclusions:**

The Knee-VEDA may be a useful tool for assessing weight-bearing knee proprioception after ACLR, showing acceptable test–retest reliability and discriminative validity. After ACL reconstruction, operated-limb proprioceptive acuity was found to be reduced during the valgus task. Further validation is needed before clinical application.

**Supplementary Information:**

The online version contains supplementary material available at 10.1186/s43019-026-00341-5.

## Background

Proprioception is a key component of the sensorimotor system, referring to the perception of body position and movement based on information from muscles, joints, skin, and other receptors [1–3]. The anterior cruciate ligament (ACL) contributes to knee stability and contains mechanoreceptors that transmit information about knee position and movement to the central nervous system [4–6]. ACL injury is a common sporting injury, and rehabilitation following ACL reconstruction (ACLR) is often complex and prolonged [7]. Because restoration of sports function requires effective integration of proprioceptive input and motor output [1], damage to ACL mechanoreceptors may impair proprioception, reduce performance, and increase the risk of re-injury [8,9]. Although ACLR is thought to restore mechanical stability and potentially improve proprioception [10], proprioceptive function after ACLR remains incompletely understood, partly owing to the limited ecological validity of current assessment methods [11].

Current proprioception assessments have three main limitations. First, the most commonly used tests, joint position sense (JPS) and threshold to detect passive motion (TTDPM), primarily assess sagittal-plane knee flexion–extension, whereas ACL injuries are typically associated with dynamic knee valgus movements in the coronal plane [11–13]. Because knee valgus requires coordinated control of the hip, knee, and ankle, these tasks may not adequately reflect the movement demands associated with ACL injury. Second, JPS and TTDPM are performed at slow, controlled speeds that differ from the self-selected speeds typical of sporting activities. Third, these assessments are generally conducted under nonweight-bearing or partial weight-bearing conditions. Although Muaidi et al. [14] assessed knee proprioception in the transverse plane, their method remained non-weight-bearing. Consequently, existing assessments may not fully capture the proprioceptive demands of weight-bearing, coronal-plane movements relevant to ACL injury.

These limitations have also restricted understanding of neuromuscular control after ACLR because muscle activation has not been assessed concurrently with proprioception during a weight-bearing, coronal-plane task. Although abnormal muscle activation has been reported in ACL-injured individuals during various movements [15–17], no study has simultaneously examined proprioceptive discrimination and neuromuscular activity during active knee valgus. Such an approach may provide insight into sensorimotor deficits after ACLR and identify potential rehabilitation targets. Accordingly, we developed a weight-bearing knee valgus proprioception assessment tool (Knee Valgus Extent Discrimination Assessment, Knee-VEDA) that enables simultaneous evaluation of coronal-plane proprioceptive discrimination and lower-limb muscle activity using surface electromyography (sEMG).

The objectives of this study were to: (1) examine the test–retest reliability and discriminative validity of the Knee-VEDA; (2) compare knee valgus proprioception and muscle activity between individuals with ACLR and healthy controls; and (3) explore relationships between proprioception, muscle activity, dynamic balance, and patient-reported outcomes post-ACLR. The primary outcomes were test–retest reliability, discriminative validity, and between-group differences in proprioceptive acuity. Secondary outcomes included sEMG activity, Y-Balance performance, patient-reported outcome measures, and their associations with proprioception. We hypothesized that: (1) the Knee-VEDA would demonstrate acceptable test–retest reliability and discriminative validity; (2) the ACLR group would show impaired proprioception and altered muscle activity compared with controls; and (3) proprioceptive performance would be associated with muscle activity, dynamic balance, and patient-reported function.

## Methods

### Study design

This cross-sectional, laboratory-based study was conducted in the biomechanics laboratory of an orthopedic hospital. It consisted of two components: (1) a test–retest reliability assessment of a novel knee valgus proprioception apparatus with a minimum seven-day interval between testing sessions; and (2) a case–control comparison evaluating knee valgus proprioception, lower-limb muscle activity, dynamic postural stability, and patient-reported functional outcomes.

### Participants

Sample size estimation was based on the study by Ciceklidag et al. [4], which reported a large effect size (Cohen’s *d* > 0.80) for proprioceptive differences between patients undergoing and healthy controls. Using *d* = 0.80, α = 0.05, and power = 0.80, the required sample size was 21 participants per group. A total of 63 participants (32 patients undergoing ACLR and 31 healthy controls) were ultimately included, exceeding this requirement. Inclusion criteria for ACLR participants were: (1) ≥ 4 months post unilateral ACL reconstruction, (2) no history of other major lower-limb injury or surgery, (3) age 18–50 years, (4) ability to perform daily activities, and (5) Tegner score > 4. Healthy controls had no history of lower-limb injury or symptoms and were matched for age (18–50 years). Thirty-six (21 ACLR, 15 controls) participants completed the retest after an interval of at least seven days. A participant flow diagram is provided in Supplementary Fig. S1. This study was approved by the Ethics Committee of the local hospital. All participants provided written informed consent prior to participation.

### Knee-VEDA test

**Fig. 1:**
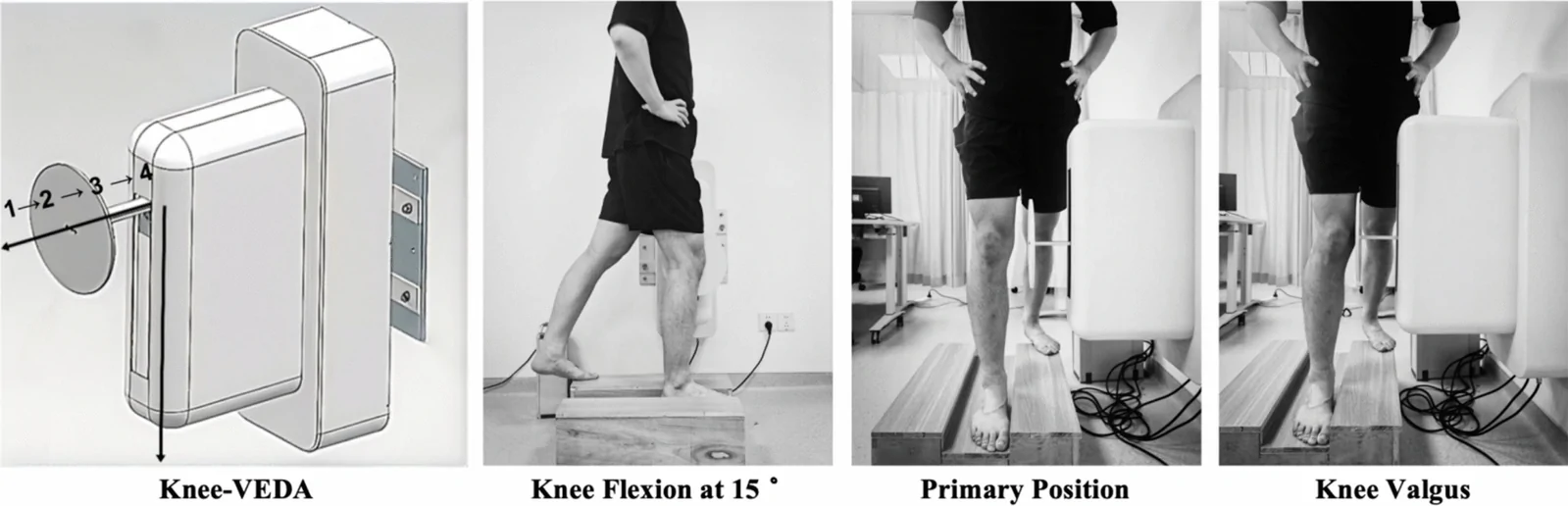
Knee-VEDA equipment and test procedure

The knee valgus extent discrimination apparatus (Knee-VEDA) was designed using mechanical regulation and automation technology. The device (30 × 50 × 80 cm, length × width × height) was wall-mounted and featured a movable disk controlled by two motors: one adjusted vertical height to accommodate individual leg length, and the other translated the disk medially and laterally to four predetermined positions (5.0, 5.7, 6.4, and 7.1 cm relative to the medial tibial condyle). During testing, participants actively moved the knee medially until the medial tibial condyle contacted the disk, thereby interacting with one of four reproducible displacement positions corresponding to distinct knee valgus magnitudes (Fig. 1). Knee valgus angles (θ) were quantified considering the lunge posture: θ ≈ arcsin (d / (L⋅cos15°)). Here, d is the lateral disk displacement, L is the leg length (35–45 cm), and L⋅cos (15°) represents the effective lever arm projected onto the frontal plane at 15° knee flexion. The resulting valgus range is 5.2–10.1°, which corresponds to the functionally relevant range reported in ACL injury literature [18,19]. The interval between adjacent positions (0.7 cm) was determined in pilot testing to avoid floor and ceiling effects.

Participants stood in a lunge position on a custom platform parallel to the Knee-VEDA, with the testing knee flexed at 15°. The device was adjusted to the height of the medial tibial condyle. Participants were instructed to look forward to minimize visual feedback, with both hands placed on the waist. During testing, participants maintained 15° knee flexion under weight-bearing conditions and actively moved the knee medially until the medial tibial condyle contacted the movable disk (Fig. 1). This movement pattern reflects a commonly reported mechanism of ACL injury [11,20]. Knee flexion angle was continuously monitored by an examiner.

The test had two phases. The first was a standardized familiarization phase, where participants completed three practice rounds of the four valgus positions (from the smallest “1” to the largest knee valgus position “4”; 12 trials in total). The second phase was formal testing. The four valgus positions were presented in random order, 5 times for each position, 20 trials in total [21]. On each trial, participants were asked to give an immediate verbal response—position 1, 2, 3, or 4—after they touched the disk and moved their knee back to the neutral starting position [3]. No feedback with respect to the correctness of the judgement was provided during the test.

Knee valgus proprioceptive acuity was quantified as the Knee-VEDA Score, defined as the mean discrimination-curve area across adjacent valgus positions [3]. The abbreviation AUC was reserved exclusively for ROC analysis.

### Muscle activity

During the proprioception testing, surface electromyography (sEMG) signals were continuously recorded using a wireless system (Noraxon Desktop DTS, Noraxon USA Inc., Scottsdale, AZ, USA) at a sampling rate of 1500 Hz. Skin preparation and electrode placement followed SENIAM guidelines [22]. Bipolar Ag/AgCl electrodes were placed over the muscle belly along the fiber direction, with an inter-electrode distance of approximately 2 cm, and secured with adhesive tape to minimize movement artifacts. sEMG signals were recorded from seven muscles involved in knee valgus control: vastus lateralis (VL), rectus femoris (RF), vastus medialis (VM), gluteus medius (GMed), gluteus maximus (GMax), semitendinosus (ST), and gastrocnemius (Gas). These muscles collectively contribute to knee extension, knee flexion control, and frontal-plane knee stability, while hip and ankle musculature play important roles in regulating dynamic knee valgus and mitigating ACL injury risk.

The EMG analysis window was defined from the onset of the first knee valgus movement to the completion of the final trial, including the return to the neutral position. Because the Knee-VEDA apparatus lacks an electronic trigger-out function, trial-by-trial EMG segmentation was not possible; therefore, a single continuous analysis window encompassing all 20 trials was used. As the Knee-VEDA system does not provide a hardware trigger, synchronized video recordings were used to identify movement onset and termination. Two investigators independently reviewed the recordings to determine the onset of the first valgus movement and the end of the final trial, ensuring that the analysis window captured the entire task while excluding preparatory activity [23]. EMG signals were processed using standard procedures. Raw signals were DC-corrected and band-pass filtered (20–450 Hz) using a fourth-order zero-phase Butterworth filter. To reduce inter-individual variability, signals were normalized to the 99th percentile (P99) of the envelope across all valid trials. Maximum voluntary contraction normalization was not used, as the task involved low-load sensorimotor activity. For time-domain feature extraction, root mean square (RMS) and mean rectified amplitude (mean amplitude) were calculated from the P99-normalized signals [24]. Frequency-domain indices and transient peak measures were not analyzed to minimize potential confounding effects from short-duration movements, noise, and inter-participant variability in task timing. The EMG signal processing pipeline is illustrated in Supplementary Fig. S2.

### Y-Balance

The Y-Balance Test was performed using the Y Balance Test Kit™ (Perform Better, West Warwick, RI, USA). Participants placed both hands on the hips and stood on one leg on the center platform, with the contralateral second toe aligned to the anterior reference line. The testing limb reached as far as possible in the anterior, posteromedial, and posterolateral directions, sliding the indicator block without losing balance or support. Trials with errors were repeated [25]. Participants performed one practice trial followed by three valid trials for each direction. A composite score was calculated as the mean reach distance across the three directions, normalized to leg length and expressed as a percentage, and used for statistical analysis.

### Self-reported outcomes

All ACLR participants completed self-report questionnaires assessing knee function, symptoms, and physical activity. The International Knee Documentation Committee (IKDC) questionnaire was used to evaluate symptoms, functional limitations, and overall knee function [26]. The Knee injury and Osteoarthritis Outcome Score (KOOS) assessed pain, symptoms, activities of daily living, sport and recreation function, and knee-related quality of life [27]. The Tegner Activity Scale was used to quantify activity level ranging from daily activities to competitive sports [28]. The Baecke Questionnaire assessed habitual physical activity across work, sport, and leisure domains [29].

### Statistical analysis

All statistical analyses were performed using R (version 4.18; R Foundation for Statistical Computing). Following Shapiro–Wilk normality testing, data were summarized as means ± SD or medians. For proprioceptive acuity, linear mixed-effects models (LMMs) were used to examine condition (ACLR-operated limb, ACLR-intact limb, and healthy control limb) and limb effects, with relevant demographic and clinical variables included as covariates and participant ID entered as a random intercept. Exploratory analyses examined the effects of sex and graft type within the ACLR cohort. Degrees of freedom were estimated using the Kenward–Roger approximation. Post hoc pairwise comparisons were performed using estimated marginal means with Benjamini–Hochberg false discovery rate (FDR) correction. Partial eta squared (η^2^_p_) was reported for fixed effects, and standardized effect sizes (Cohen’s d) with corresponding 95% confidence intervals (CIs) were reported for pairwise comparisons. The same LMM framework was applied to sEMG outcomes. To account for multiple testing across muscles, FDR correction was applied separately for between-group and within-group comparison families. Independent samples *t*-tests were used to compare demographic characteristics (age, height, and body mass) and Y-Balance performance between groups, with Cohen’s *d* and 95% CIs reported as effect size measures. For healthy controls, the dominant (preferred kicking) limb was used for primary comparisons. Discriminative validity was evaluated using receiver operating characteristic (ROC) curve analysis, with the area under the curve (AUC) and 95% CIs reported. Test–retest reliability was assessed using the intraclass correlation coefficient (ICC_3,1_), standard error of measurement (SEM), minimal detectable change (MDC), and Bland–Altman analysis. Pearson correlation coefficients were used to examine associations among proprioceptive acuity, clinical outcomes, and sEMG variables. Statistical significance was set at an FDR-adjusted *p* < 0.05.

## Results

Table 1 summarizes demographic characteristics, proprioceptive acuity, and self-reported scores in the ACLR and control groups. Groups did not differ in age (Cohen’s *d* =  −0.13, 95% CI [−0.63, 0.36], _p_ = 0.60) or height (Cohen’s *d* =  −0.28, 95% CI [−0.77, 0.22], _p_ = 0.28), whereas body mass was significantly higher in the ACLR group (Cohen’s *d* =  −0.62, 95% CI [−1.13, −0.12], _p_ = 0.02). All participants had Tegner scores ≥ 4. For dynamic balance, the Y-Balance composite score was significantly lower in the ACLR group compared with controls (Cohen’s *d* = 0.58, 95% CI [0.09, 1.07], _p_ = 0.02).

**Table 1:** Participant Characteristics and results of knee proprioception

	Healthy (*n* = 31)	ACLR (*n* = 32)
Age (yrs)	26.32 ± 2.61	26.88 ± 5.34
Height (cm)	171.71 ± 8.58	174.09 ± 8.69
Body mass (kg)	63.89 ± 10.24	71.77 ± 14.62*
Mean time since surgery (mo)		24.9 ± 17.0
Knee-VEDA Score		
Healthy dominant side	0.787 ± 0.050	–
ACL operated side	–	0.701 ± 0.075**^#^
ACL intact side	–	0.766 ± 0.065
Male (12;18)	0.789 ± 0.067	0.697 ± 0.084**
Female (19;14)	0.784 ± 0.041	0.706 ± 0.063**
AL (n = 11)		0.702 ± 0.059
Autograft (*n* = 21)		0.700 ± 0.083
Y-Balance	102.85 ± 10.17	96.53 ± 10.23*
Tegner score	7 [6,8]	6 [5,7]
KOOS		
Symptoms	–	75.22 ± 10.88
Pain	–	89.41 ± 8.03
ADL	–	96.69 ± 3.72
SRF	–	80.00 ± 19.67
QoL	–	61.52 ± 21.01
IKDC	–	83.28 ± 7.48
Baecke	–	36.59 ± 5.52

^**^ = significantly different from healthy dominant side (*p* < 0.001)

^*^ = significantly different from healthy dominant side (*p* < 0.05)

^#^ = significantly different from ACL-intact side (*p* < 0.01)

*ACLR* anterior cruciate ligament reconstruction, *Knee-VEDA Score* mean area under the discrimination curve across adjacent valgus positions, *AL* artificial ligament, *KOOS* knee injury and osteoarthritis outcome score, *ADL* activities of daily living, *SRF* sport and recreation function, *QoL* quality of life, *IKDC* International Knee Documentation Committee

**Fig. 2:**
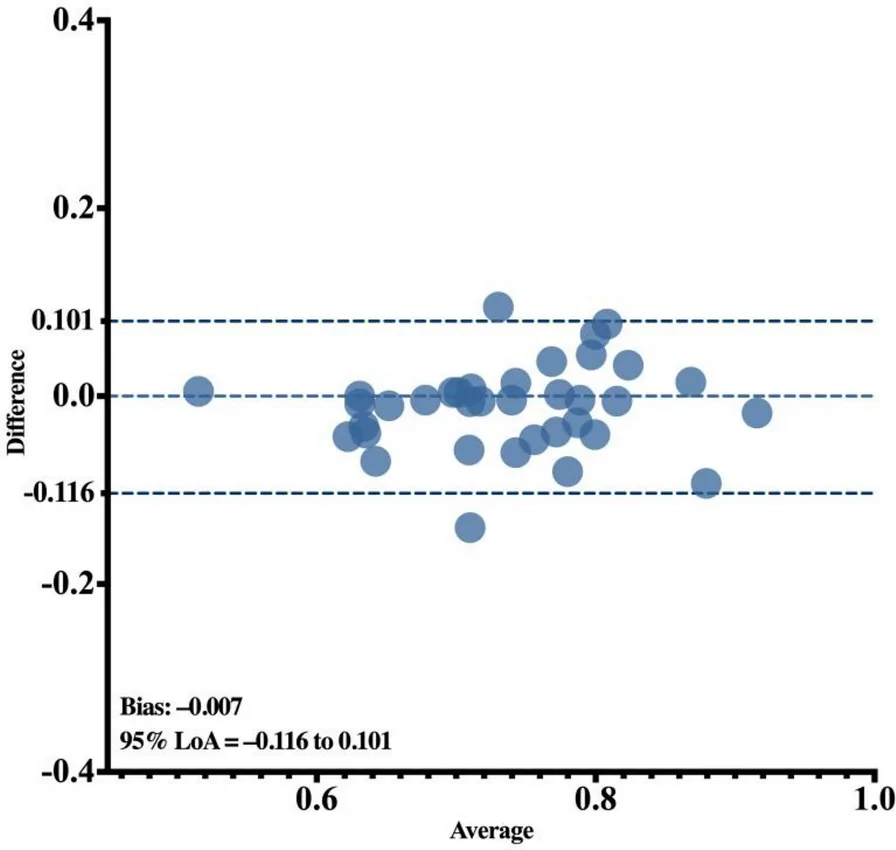
Bland–Altman plots of the scores from the two sessions. *LoA*,limits of agreement

Overall test–retest reliability was good (ICC_3,1_ = 0.85), with a low coefficient of variation (CV = 2.38 ± 2.77%). The SEM (0.007) and MDC (0.019) were small, indicating minimal measurement error (Table 2). Bland–Altman analysis revealed a negligible bias (–0.007) with narrow 95% limits of agreement (–0.116 to 0.101), supporting the consistency of the measurements (Fig. 2). However, subgroup analysis showed good reliability in the ACLR group and moderate, imprecise reliability in healthy controls.

**Table 2:** Reliability data of knee proprioception test

	Session 1	Session 2	ICC (95%CI)	CV	MDC	SEM
Total (*n* = 36)	0.732 ± 0.089	0.741 ± 0.084	0.85 (0.73, 0.92)	2.38 ± 2.77	0.019	0.007
Healthy (*n* = 15)	0.795 ± 0.055	0.799 ± 0.068	0.64 (0.20, 0.86)	2.44 ± 2.21	0.038	0.014
ACLR (*n* = 21)	0.698 ± 0.074	0.703 ± 0.063	0.84 (0.65, 0.93)	2.36 ± 2.69	0.021	0.008

*ACLR* anterior cruciate ligament reconstruction, *CV* coefficient of variation, *ICC* intraclass correlation coefficient, *SEM* standard error of measurement, *MDC* minimal detectable change

**Fig. 3:**
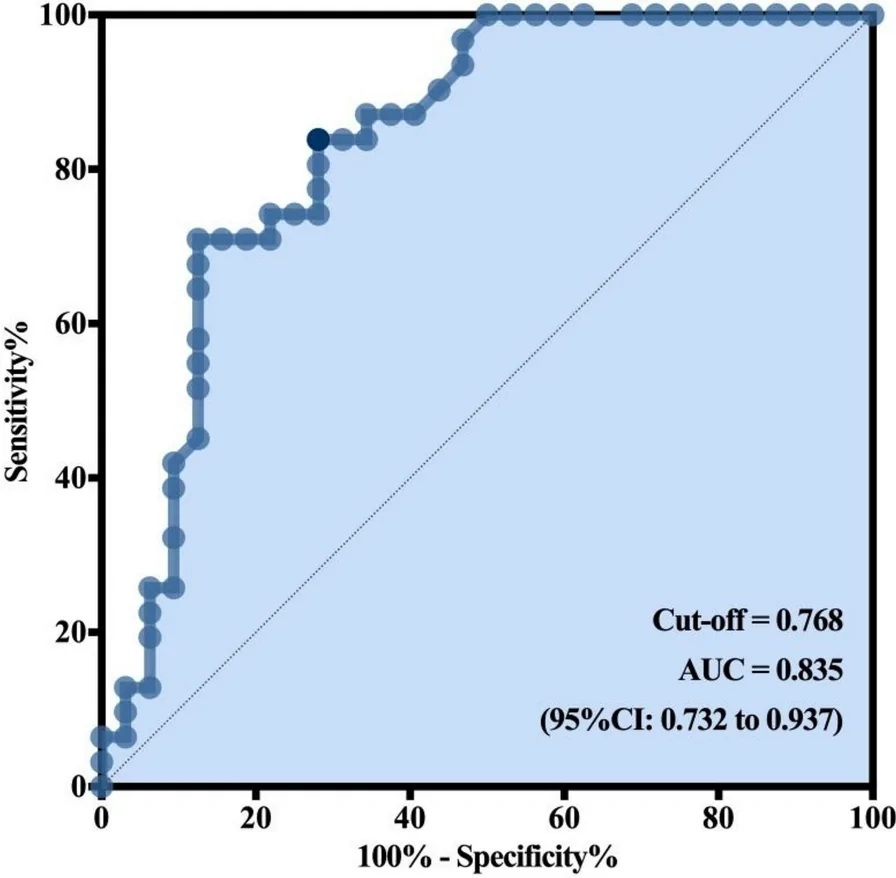
ROC curve for the knee valgus proprioception test as an ACLR detector

ROC analysis showed that the Knee-VEDA demonstrated good discriminative ability between the ACLR and control groups, with an AUC of 0.835 (95% CI [0.73, 0.94], *p* < 0.001). The optimal cutoff value was 0.768, with a sensitivity of 0.719 (95% CI [0.54, 0.85]) and specificity of 0.879 (95% CI [0.78, 0.95]) (Fig. 3).

LMM analysis revealed a significant main effect of condition on knee valgus proprioceptive acuity (F = 19.87, *p* < 0.001, η^2^_p_ = 0.43). Post hoc comparisons showed that the ACLR-operated limb exhibited significantly lower proprioceptive acuity than both healthy controls (Cohen’s *d* = 1.47, 95% CI [0.63, 2.31], *p* < 0.001) and the ACLR-intact limb (Cohen’s *d* = 1.52, 95% CI [0.97, 2.07], *p* < 0.001), whereas no difference was observed between the ACLR-intact limb and healthy controls (Cohen’s *d* = 0.05, 95% CI [− 0.86, 0.77], _p_ = 0.91).

Within the ACLR cohort, a significant main effect of testing limb was observed (F = 36.08, *p* < 0.001, η^2^_p_ = 0.54), with the operated knee demonstrating poorer proprioceptive acuity than the intact knee (Cohen’s *d* = 1.52, 95% CI [0.97, 2.07], *p* < 0.001). No significant effects of postoperative duration (_p_ = 0.57), leg length (_p_ = 0.88), body mass (_p_ = 0.53), sex (_p_ = 0.70), or graft type (_p_ = 0.94) were identified.

**Fig. 4:**
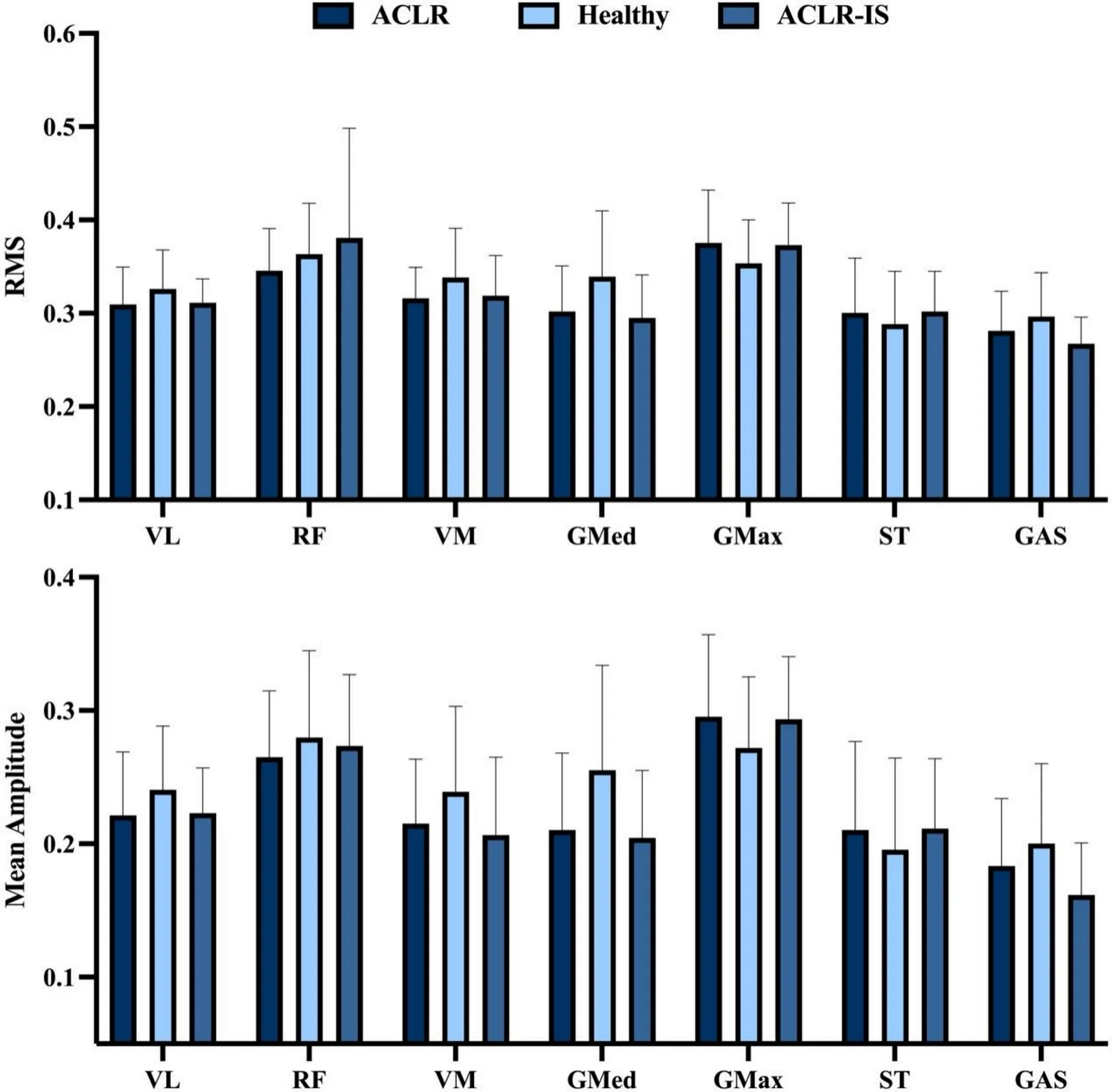
The difference in muscle activity during the knee valgus proprioception test. *ACLR-IS* intact side of anterior cruciate ligament reconstruction people, *RMS* root mean square, *VL* vastus lateralis, *RF* rectus femoris, *VM* vastus medialis, *GMed* gluteus medius, *GMax* gluteus maximus, *ST* semitendinosus, *GAS* gastrocnemius, *FDR* false discovery rate

Figure 4 presents sEMG activity during the proprioceptive discrimination task. No significant between-group or between-limb differences were observed after FDR correction (Supplementary Table S2). In healthy controls, proprioceptive acuity was significantly correlated with vastus medialis mean amplitude (_r_ = 0.53, adjusted _p_ = 0.002), whereas no significant associations were observed in the ACLR group (_p_ = 0.14–0.96). Time since surgery was not correlated with any sEMG outcomes (_r_ =  −0.04 to 0.07, _p_ = 0.66–0.82).

In the ACLR group, surgical side knee valgus proprioception was significantly correlated with Y-Balance Test performance (_r_ = 0.50, *p* < 0.001), and the KOOS Symptoms sub-scale (_r_ = 0.55, _p_ = 0.001). No significant correlations were observed with other outcome measures.

## Discussion

This study assessed knee proprioception during a weight-bearing, coronal-plane valgus discrimination task with simultaneous sEMG in individuals with ACLR. The main findings were: (1) The Knee-VEDA demonstrated good test–retest reliability and acceptable discriminant validity; (2) ACLR participants showed significantly poorer proprioceptive acuity on the operated knee compared with both healthy controls and their intact limb; (3) Knee-VEDA proprioceptive discrimination scores in ACLR participants were significantly correlated with Y-Balance performance and KOOS symptoms; and (4) lower-limb muscle activity did not differ significantly between groups after multiple-comparison correction, whereas a significant association between knee valgus proprioception and VM activity was uniquely observed in healthy controls.

The results demonstrated that the Knee-VEDA exhibited good test–retest reliability and acceptable discriminative validity in assessing proprioceptive function in ACLR participants. This suggests that the test is reliable and effective in identifying proprioceptive impairments, consistent with previous findings using the active movement extent discrimination assessment [30,31]. Although Muaidi et al. [14] previously investigated knee rotation proprioception, their assessments were performed in a seated position. By comparison, the Knee-VEDA is more relevant to functional and sport-specific movements. These findings indicate that the Knee-VEDA is a promising tool for assessing knee proprioception in individuals after ACLR. However, the lower test–retest reliability in healthy controls compared with the ACLR group may reflect a ceiling effect, as healthy participants performed near the upper limit of the task, restricting score variability and suppressing the ICC. However, the relatively small retest sample may also have contributed to the modest reliability estimate. This suggests that the Knee-VEDA is better suited for detecting proprioceptive deficits in clinical populations, and further evaluation in larger healthy samples is warranted before conclusions regarding its utility in uninjured individuals can be drawn.

The results showed that in ACLR participants, proprioceptive acuity of the operated knee was significantly poorer than that of both healthy controls and the intact limb, whereas the intact limb did not differ significantly from controls. These findings are consistent with previous research reporting impaired knee proprioception after ACL injury and reconstruction [5,8,11]. These results indicate that despite ACLR, proprioceptive input from the operated knee remains impaired, suggesting that surgery may not fully restore it, and this should be addressed in rehabilitation programs.

To the best of our knowledge, this is the first study to simultaneously assess knee valgus proprioception and EMG activity. Previous studies have shown that ACLR individuals exhibit greater knee valgus during landing and cutting tasks [32,33]. By contrast, the present study required participants to actively perform knee valgus movements, enabling evaluation of neuromuscular activation during both movement execution and proprioceptive perception. ACLR participants showed reduced intact-limb RMS activity of GMed and GAS compared with healthy controls at the uncorrected level; however, these differences did not remain significant after FDR correction. For mean amplitude, several between-group differences were observed at the uncorrected level, but none survived multiple-comparison correction. In healthy controls, knee valgus proprioception was significantly associated with vastus medialis activity, whereas this relationship was not observed in the ACLR group. This may indicate altered sensorimotor integration following ACL reconstruction; however, the finding should be interpreted cautiously owing to the cross-sectional design, limited sample size, inter-individual variability, and the broad EMG analysis window. Further studies with larger cohorts and more comprehensive neuromuscular assessments are needed to clarify the relationship between proprioception and muscle activation after ACL reconstruction.

In ACLR participants, Knee-VEDA scores were significantly correlated with Y-Balance Test performance, indicating an association between knee valgus proprioception and dynamic postural control. This may be partly explained by the weight-bearing nature of the Knee-VEDA, which imposes sensorimotor demands that overlap with those of dynamic balance tasks. Proprioceptive performance was also significantly correlated with the KOOS Symptoms subscale, suggesting that poorer knee valgus proprioception is associated with greater subjective symptom severity. Given the weight-bearing, movement-based nature of the task, the sensory function assessed by the Knee-VEDA may be relevant to the perception of symptoms such as pain, stiffness, or swelling during daily activities after ACLR. Overall, these findings suggest that reduced knee valgus proprioceptive acuity is associated with impaired dynamic balance and worse patient-reported symptoms, underscoring its functional relevance following ACL reconstruction.

The findings of this study have several implications for ACLR rehabilitation and training. First, Han et al. have suggested that proprioceptive impairment may be task-specific [30], the knee valgus proprioceptive deficits identified here may be most effectively addressed through rehabilitation exercises that incorporate coronal-plane, weight-bearing movement discrimination, rather than sagittal-plane training alone. Second, in uninjured individuals, these findings may also highlight the potential value of neuromuscular training targeting hip and lower-limb musculature during dynamic knee valgus tasks, which may help optimize movement control during sport-related activities. Finally, because between-group sEMG differences did not remain significant after FDR correction, rehabilitation implications should be interpreted primarily from the proprioceptive and dynamic balance findings. Future studies using trial-wise EMG segmentation are needed before neuromuscular training implications can be drawn.

This study has several limitations that should be acknowledged. First, knee valgus angles were not directly measured but were estimated using a simplified geometric model, and participants used self-selected movement strategies. While this preserved ecological validity, the geometric model assumes pure coronal-plane rotation and does not account for tibial rotation, hip adduction, or soft tissue deformation, and variability in movement patterns may have introduced unmeasured kinematic differences. Future studies should incorporate motion capture for direct kinematic quantification. Second, several EMG-related limitations should be noted. The analysis window spanned the entire multi-trial task because the Knee-VEDA apparatus lacks an electronic trigger-out function, precluding trial-by-trial segmentation; the resulting measures therefore represent overall task-level muscle activity rather than phase-specific activation. Third, the ACLR group exhibited substantial heterogeneity in postoperative time (minimum 4 months since surgery), and detailed surgical information, including graft harvest site, concomitant meniscal or chondral injury, rehabilitation stage, and return-to-sport status, was not systematically available. This limits the interpretation of sEMG findings and precludes stratified analyses by recovery phase. Future studies should employ prospective designs with larger, sex-balanced, and more homogeneous postoperative cohorts, complete surgical documentation, and direct kinematic measurement.

## Conclusions

The Knee-VEDA showed promising test–retest reliability and discriminative validity in a laboratory setting. After ACL reconstruction, operated-limb proprioceptive discrimination was impaired. These findings support further validation and longitudinal study before routine clinical implementation.

## Supplementary Information

Additional file 1.

## Data Availability

The datasets used and/or analyzed during the current study are available from the corresponding author on reasonable request.
