# Supplementary material for: Knee valgus proprioception, muscle activity, patient-reported outcomes and dynamic balance control after anterior cruciate ligament reconstruction: a cross-sectional case–control study
Source: Knee Surg Relat Res. 2026 Aug 20;38:43. doi: 10.1186/s43019-026-00341-5 (PMC13491738; doi:10.1186/s43019-026-00341-5)
Supplement: Supplementary file 1 [file 43019_2026_341_MOESM1_ESM.docx]

Supplementary Figure S1. Participant flow diagram.

Sixty-three participants (32 ACLR, 31 controls) were enrolled and completed the main test session. A subset of 36 participants (21 ACLR, 15 controls) returned for retest after a minimum 7-day interval for test–retest reliability analysis. PROMs = patient-reported outcome measures (IKDC, KOOS, Tegner, Baecke), completed by the ACLR group only.

Supplementary Figure S2. Flowchart of EMG Signal Preprocessing and Feature Extraction Pipeline.

Abbreviation: EMG, Electromyography; fs, Sampling Frequency; HZ, Hertz; abs, Absolute value; P99, the 99th percentile; sqrt, Square Root; RMS, Root Mean Square.

Supplementary Table S2. Between-group and within-group comparisons of sEMG activity during the Knee-VEDA task.

| Variable | Muscle | Comparison | Cohen's d | 95% CI | Raw p | Adjusted p |
| --- | --- | --- | --- | --- | --- | --- |
| RMS | GMed | ACLR intact vs Control | 0.81 | [0.23, 1.39] | 0.006 | 0.13 |
| RMS | GAS | ACLR intact vs Control | 0.70 | [0.14, 1.25] | 0.014 | 0.14 |
| RMS | VM | ACLR intact vs Control | 0.54 | [-0.16, 1.24] | 0.132 | 0.29 |
| RMS | GMax | ACLR intact vs Control | 0.38 | [-0.89, 0.12] | 0.138 | 0.29 |
| RMS | VL | ACLR intact vs Control | 0.37 | [-0.15, 0.88] | 0.168 | 0.32 |
| RMS | RF | ACLR intact vs Control | 0.29 | [-0.84, 0.27] | 0.314 | 0.50 |
| RMS | ST | ACLR intact vs Control | 0.25 | [-0.75, 0.26] | 0.334 | 0.50 |
| RMS | GMed | ACLR operated vs Control | 0.67 | [0.09, 1.25] | 0.023 | 0.16 |
| RMS | VM | ACLR operated vs Control | 0.62 | [-0.09, 1.31] | 0.086 | 0.29 |
| RMS | GMax | ACLR operated vs Control | 0.43 | [-0.94, 0.07] | 0.094 | 0.29 |
| RMS | VL | ACLR operated vs Control | 0.42 | [-0.10, 0.94] | 0.113 | 0.29 |
| RMS | GAS | ACLR operated vs Control | 0.32 | [-0.23, 0.86] | 0.257 | 0.45 |
| RMS | ST | ACLR operated vs Control | 0.23 | [-0.73, 0.28] | 0.380 | 0.53 |
| RMS | RF | ACLR operated vs Control | 0.20 | [-0.35, 0.76] | 0.472 | 0.62 |
| Mean Amplitude | GMed | ACLR intact vs Control | 0.89 | [0.26, 1.51] | 0.006 | 0.09 |
| Mean Amplitude | VM | ACLR intact vs Control | 0.83 | [0.10, 1.55] | 0.026 | 0.14 |
| Mean Amplitude | GAS | ACLR intact vs Control | 0.74 | [0.19, 1.29] | 0.008 | 0.09 |
| Mean Amplitude | GMax | ACLR intact vs Control | 0.39 | [-0.89, 0.12] | 0.132 | 0.31 |
| Mean Amplitude | VL | ACLR intact vs Control | 0.38 | [-0.17, 0.92] | 0.176 | 0.37 |
| Mean Amplitude | ST | ACLR intact vs Control | 0.24 | [-0.74, 0.26] | 0.345 | 0.57 |
| Mean Amplitude | RF | ACLR intact vs Control | 0.08 | [-0.52, 0.68] | 0.796 | 0.93 |
| Mean Amplitude | GMed | ACLR operated vs Control | 0.77 | [0.15, 1.39] | 0.015 | 0.11 |
| Mean Amplitude | VM | ACLR operated vs Control | 0.61 | [-0.11, 1.33] | 0.095 | 0.31 |
| Mean Amplitude | GMax | ACLR operated vs Control | 0.42 | [-0.93, 0.08] | 0.102 | 0.31 |
| Mean Amplitude | VL | ACLR operated vs Control | 0.42 | [-0.12, 0.96] | 0.127 | 0.31 |
| Mean Amplitude | GAS | ACLR operated vs Control | 0.27 | [-0.26, 0.81] | 0.319 | 0.57 |
| Mean Amplitude | ST | ACLR operated vs Control | 0.23 | [-0.73, 0.27] | 0.374 | 0.57 |
| Mean Amplitude | RF | ACLR operated vs Control | 0.26 | [-0.35, 0.86] | 0.406 | 0.57 |

Abbreviations: RMS, root mean square; GMed, gluteus medius; GAS, gastrocnemius; VM, vastus medialis; GMax, gluteus maximus; VL, vastus lateralis; RF, rectus femoris; ST, semitendinosus.
